# Intratumoral Microbiome of Metastatic Pancreatic Ductal Adenocarcinoma

**DOI:** 10.3390/ijms27104210

**Published:** 2026-05-09

**Authors:** Vladislav Pavlov, Anastasiya Snezhkina, Elena Pudova, Marina Emelyanova, Elena Fedoseeva, Alyona Filatova, Dmitry Kalinin, Anna Kudryavtseva, Maria Fedorova

**Affiliations:** 1 Engelhardt Institute of Molecular Biology, Russian Academy of Sciences, 119991 Moscow, Russia; vladislav1pavlov@gmail.com (V.P.); leftger@rambler.ru (A.S.); pudova_elena@inbox.ru (E.P.); emel_marina@mail.ru (M.E.); elfed0@mail.ru (E.F.); rhizamoeba@mail.ru (A.K.); 2 Vishnevsky Institute of Surgery, Ministry of Health of the Russian Federation, 117997 Moscow, Russia; alyonafilatova17@gmail.com (A.F.); dmitry.v.kalinin@gmail.com (D.K.)

**Keywords:** PDAC, microbiome, 16s sequencing, metastasis, KRAS

## Abstract

Pancreatic ductal adenocarcinoma (PDAC) remains one of the most lethal oncological diseases, with a 5-year survival rate of approximately 13%—among the lowest in oncology. Poor survival is driven by aggressive tumor progression and metastasis, which may be influenced by the tumor microbiome. This study aimed to evaluate the role of microbiome in PDAC progression and metastasis. First, we assessed the microbial composition of control samples (surface swabs, empty paraffin, extraction controls, and sequencing controls) and removed contaminant taxa. Overall bacterial biomass was extremely low, with no significant differences in alpha or beta-diversity between tumor and normal tissue. *Kocuria rosea* was significantly enriched in tumors compared to normal tissue, and this difference persisted after decontamination. Metastatic tumors showed altered abundance of *K. rosea* and *Herbaspirillum huttiense*, whereas non-metastatic tumors differed in *Lysobacter bugurensis*, *Caulobacter ginsengisoli*, and *H. huttiense* relative to normal tissue. No global compositional differences were observed between KRAS-mutant and wild-type tumors; however, KRAS-mutant tumors exhibited differential enrichment of *K. rosea* and *L. bugurensis* relative to adjacent normal tissue. The PDAC microbiome harbors very low bacterial biomass and does not robustly distinguish tumor from normal tissue at the community level. Nonetheless, *K. rosea* emerges as a candidate taxon differentially enriched in PDAC, with potential stage- and KRAS-associated patterns. These findings highlight the need for orthogonal validation (qPCR, FISH, culture) and larger prospective cohorts to differentiate true biological associations from residual contamination or stochastic noise in low-biomass settings.

## 1. Introduction

Pancreatic ductal adenocarcinoma is one of the most aggressive and deadly forms of cancer with an overall 5-year survival rate of 13% [1]. Despite advances in oncology, the five-year survival rate for patients with metastatic disease remains critically low [2]. This rate does not exceed 3%, and the median survival without radical surgery is less than one year. By 2030, this pathology is projected to become the second leading cause of cancer death in developed countries. This data underscores the urgent need to find new biological targets and therapeutic strategies [3,4,5,6]. In the last decade, researchers have turned their attention to a previously underestimated component of the tumor microenvironment: the microbiome. For a long time, it was believed that internal organs such as the pancreas were sterile, but pioneering research in recent years has disproved this dogma. It has been found that pancreatic tumors have a specific microbial composition that differs significantly from the microbiome of healthy tissue [7,8]. Emerging data suggest a hypothesis in which bacteria may accompany migrating tumor cells and could contribute to the formation of a pre-metastatic niche [9]. By modulating the immune response, producing specific metabolites, and directly affecting the signaling pathways of tumor cells, the microbiome may contribute to the survival, invasion, and dissemination of cancer (colorectal cancer, breast cancer, PDAC and others) [10,11,12]. PDAC cells have increased levels of certain bacteria (*Fusobacterium, Proteobacteria, Bacteroidetes*) [7,8,13].

Research on the tumor microbiome has advanced over recent years, yet interpreting findings remains difficult because of the technical challenges associated with low-biomass samples [14]. Previous clinical studies of PDAC have yielded conflicting results regarding the prevalence and oncological significance of the tumor microbiome, adjacent pancreatic tissue, and precancerous lesions [15,16]. Furthermore, they do not address the relationship between tumor invasion and metastasis and the intratumoral microbiome of PDAC.

This study aims to understand the role of the microbiome in PDAC progression and metastasis, considering a number of factors: sample contamination during surgical removal, storage-related DNA degradation, and detection of microorganisms from environmental sources during sample handling. To ensure reliable results despite low bacterial DNA and potential contamination, we combined rigorous controls with experimental measures (multiplex PCR), contaminant-aware bioinformatics (decontam) and robust statistical methods (ANCOM-BC). In this study, we seek to assess whether there are differences in the microbiome of metastatic and non-metastatic tumors, KRAS-mutant and wild-type PDAC.

## 2. Results

### 2.1. Sample Characteristics

A PDAC sample collection has been established, comprising tumor samples along with paired normal adjacent tissue (Table 1).

Among the 50 patients from whom FFPE samples were obtained and analyzed, the median age was 61 years. This cohort included 38 patients with lymph node metastases, 12 patients without metastases, and 3 with distant metastases. Patients with distant metastases were not analyzed as a separate group due to small group size (n = 3). Cases with M1 also had lymphogenous metastases and were included in the group with lymphogenous metastases (N+) for analysis. The median age across these three groups was identical (61 years), and all tumors were localized in the head of the pancreas. Various *KRAS* gene mutations were identified in 43 patients: 18 had *KRAS* G12D, 14 had *KRAS* G12V, 9 had *KRAS* G12R, 1 had *KRAS* Q61H, and 1 had *KRAS* G12S. Only seven patients exhibited no *KRAS* mutations (wild-type).

### 2.2. Microbiome Composition in Control Samples

Sequencing data were obtained from several types of negative controls: samples from various surfaces and equipment in the pathology and molecular genetics laboratories, 159 paraffin controls (paraffin sections without tissue), and blank controls from the stages of DNA extraction, PCR, and sequencing (no-template controls). These data were combined into a composite negative control.

**Figure 1:**
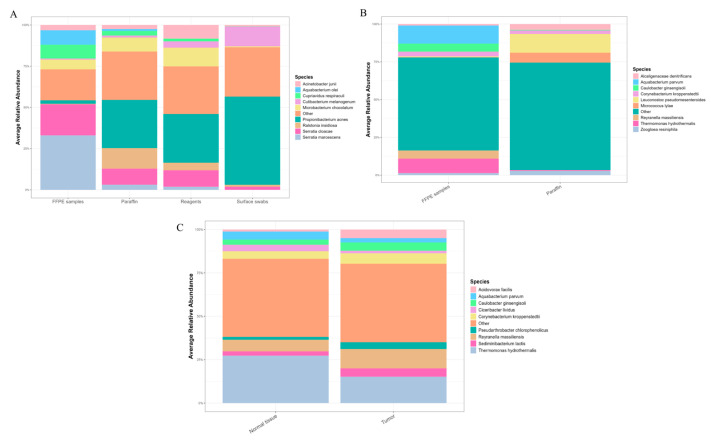
Stacked bar plot of the microbiome composition (top 10 expressed genera) in PDAC. (**A**) All microbial genera detected in PDAC, paraffin, reagents and surface swabs (top 10 from full data). (**B**) The top 10 genera in the decontaminated data (elimination of reagents and surface swab genera). (**C**) The top 10 genera in normal and tumor tissue after decontam.

The identified bacterial species and their relative abundance in the negative controls are shown in Figure 1 and Table 2.

The predominance of contaminating bacterial DNA was expected due to the low microbial biomass in the tumor samples. Species such as *Brachybacterium conglomeratum*, *Microbacterium maritypicum*, *Modestobacter multiseptatus*, *Serratia marcescens*, *Pelomonas puraquae*, *Curtobacterium herbarum*, *Propionibacterium acnes*, and *Microbacterium chocolatum* were the primary contributors to contamination in the PDAC samples (Figure 1).

### 2.3. Contaminant Filtering and Taxonomic Profiling

To obtain high-quality data reflecting the actual composition of the microbiome, a thorough decontamination pipeline was utilized. Firstly, any species detected in either extraction reagent blanks or swab controls were removed as a potential laboratory or superficial contaminant. Secondly, the decontam package [17] was used with empty paraffin samples as negative controls (prevalence method, threshold = 0.5) for the exclusion of taxa more prevalent in control samples. ASVs were aggregated at the species level; out of 3989 species, 291 (7%) were not counted as contaminants and were passed down for further analysis.

Figure 1 shows stacked bar plots of average relative abundance of bacterial species across sample types. The composition differs across FFPE samples, paraffin, reagents, surface swabs, and between normal and tumor tissues, with several taxa dominating each category. A complete list of bacterial species identified in the FFPE PDAC samples is provided in Supplementary File S1.

### 2.4. Bacterial Composition in Tumor and Adjacent Normal Tissue

**Figure 2:**
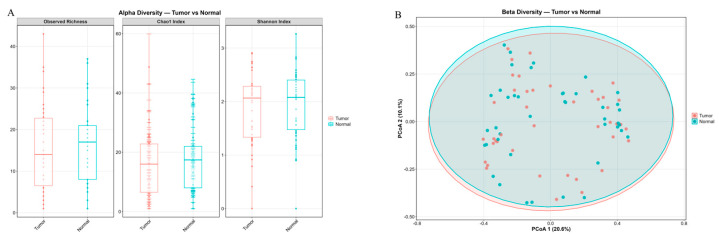
Microbial diversity in normal and tumor tissues. (**A**) Alpha-diversity indices: Observed Richness, Chao1, and Shannon for normal and tumor tissues. (**B**) Beta-diversity analyses among normal and tumor tissues. Principal Component Analysis (PCoA) plots based on Bray–Curtis dissimilarity.

Alpha-diversity was assessed in normal and tumor tissues. Analyses of alpha-diversity, as measured by the Chao1, Shannon, and Observed Richness indices, revealed no statistically significant differences between the groups (Figure 2). Furthermore, assessment of beta-diversity via principal coordinate analysis (PCoA) based on Bray–Curtis dissimilarity showed no apparent clustering of samples according to group membership. This indicates a lack of substantial divergence in the overall microbial community structure.

**Figure 3:**
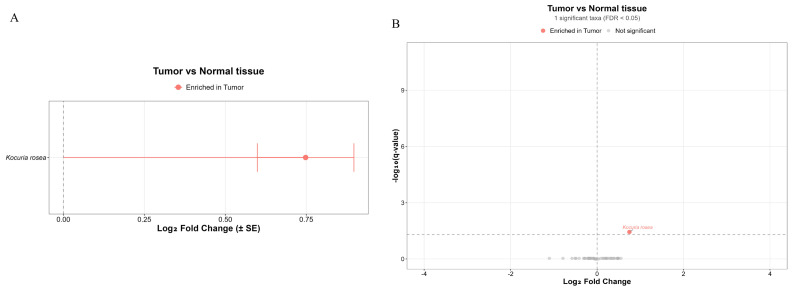
Comparison of microbiota in normal and tumor tissues. (**A**) Comparison of microbiota in normal and tumor tissues using ANCOMBC2. (**B**) Volcano plot demonstrating the differential prevalence of bacteria between normal and tumor tissues. Bacteria are colored according to the tumor type (tumor in red) if they passed significance thresholds (FDR-corrected Q value < 0.05).

Analysis of the microbiome composition in normal and tumor tissues revealed differential differences in the content of *Kocuria rosea* (Figure 3, Supplementary Figure S1).

We examined whether individual clinical/pathological variables (tumor grade, invasion status, age, sex) were associated with overall microbiome composition using Spearman correlation and Mann–Whitney tests with FDR correction. No statistically significant associations were detected. However, we did not perform multivariate adjustment for potential confounders such as FFPE block age, sequencing depth, or treatment history because these data were incomplete or not recorded. Therefore, residual confounding cannot be excluded.

### 2.5. Bacterial Composition in Metastatic and Non-Metastatic Tumors

**Figure 4:**
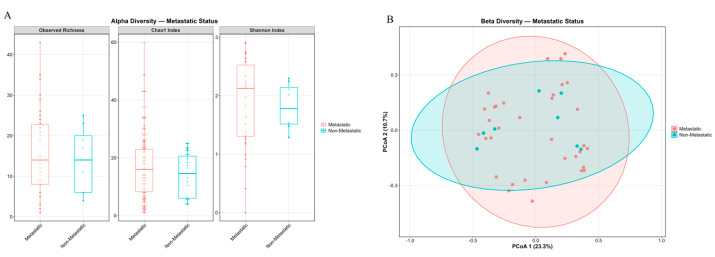
Microbial diversity in metastatic and non-metastatic PDACs. (**A**) Alpha-diversity indices: Observed Richness, Chao1, and Shannon for metastatic and non-metastatic PDAC. (**B**) Beta-diversity analyses among metastatic and non-metastatic PDAC. Principal Component Analysis (PCoA) plots based on Bray–Curtis dissimilarity.

Alpha-diversity was assessed in metastatic and non-metastatic PDAC samples. Analyses of alpha-diversity, as measured by the Chao1, Shannon, and Observed Richness indices, revealed no statistically significant differences between the groups (Figure 4). Furthermore, assessment of beta-diversity via principal coordinate analysis (PCoA) based on Bray–Curtis dissimilarity showed no apparent clustering of samples according to group membership. This indicates a lack of substantial divergence in the overall microbial community structure.

**Figure 5:**
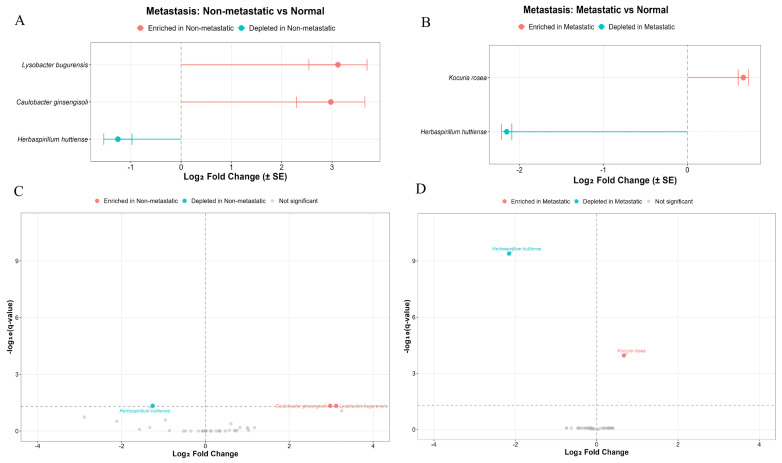
Comparison of microbiota in metastatic and non-metastatic PDAC vs. normal tissue. (**A**,**B**): Comparison of microbiota in metastatic and non-metastatic PDAC vs. normal tissue using ANCOMBC2. (**C**,**D**) Volcano plot demonstrating the differential prevalence of bacteria between non-metastatic/metastatic and normal tissue. Bacteria are colored according to the tissue type (non-metastatic (**C**) and metastatic (**D**)—red; normal tissue—blue) if they passed significance thresholds (FDR-corrected Q value < 0.05).

Analysis of the microbiome composition in metastatic and non-metastatic PDAC tumors did not reveal different bacterial contents. However, comparing the microbiota content in metastatic tumors with that in adjacent normal tissues revealed differential species content of *Kocuria rosea* and *Lysobacter bugurensis*. Similarly, comparing the microbiota content in non-metastatic tumors and adjacent normal tissues revealed differences in the presence of Lysobacter bugurensis, *Caulobacter ginsengisoli* and *Herbaspillium huttiense* (Figure 5, Supplementary Figure S1).

### 2.6. The Effect of KRAS Mutations on the Bacterial Composition of Tumors

**Figure 6:**
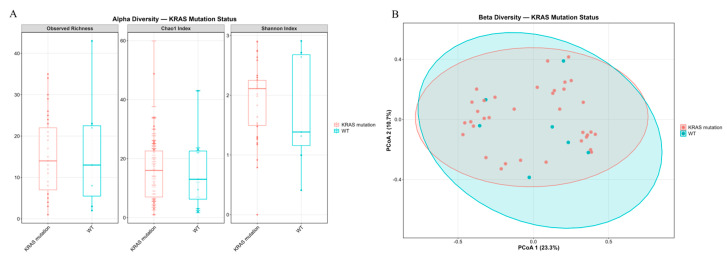
Microbial diversity in KRAS-mutant and KRAS wild-type PDACs. (**A**) Alpha-diversity indices: Observed Richness, Chao1, and Shannon for KRAS-mutant and KRAS wild-type PDAC. (**B**) Beta-diversity analysis among KRAS-mutant and KRAS wild-type PDAC. Principal Component Analysis (PCoA) plots based on Bray–Curtis dissimilarity.

The alpha-diversity of RAS-mutant and wild-type PDAC was assessed. No significant differences in the Chao1, Shannon, and Observed Richness indices were found. Analysis of the beta-diversity of RAS-mutant and wild-type PDAC revealed no significant clustering (Figure 6).

**Figure 7:**
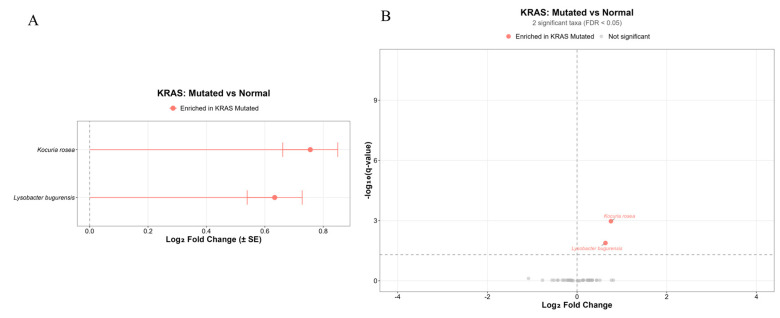
Comparison of microbiota in KRAS-mutant tumors and normal tissue. (**A**) Comparison of microbiota in KRAS-mutant PDACs and normal tissue using ANCOM2. (**B**) Volcano plot demonstrating the differential prevalence of bacteria between KRAS-mutant PDACs and normal tissue. Bacteria are colored according to the tumor type (KRAS-mutant, red) if they passed significance thresholds (FDR-corrected Q value < 0.05).

Analysis of the microbiome composition in wild-type and KRAS-mutant pancreatic tumors did not reveal differences. However, when comparing the microbiome composition of KRAS-mutant tumors with that of normal tissue, differential differences in composition were identified: *Kocuria rosea* and *Lysobacter bugurensis* (Figure 7, Supplementary Figure S1).

## 3. Discussion

Metastatic pancreatic cancer is one of the most challenging problems in modern oncology, and its microbial content may be the most unexpected and promising key to solving it. When it comes to the factors that determine the aggressiveness of PDAC, genetic mutations (e.g., in the KRAS gene) and tumor stroma characteristics are traditionally considered. Currently, attempts are being made to include a third, dynamic component: the intratumoral microbiome. The unique community of bacteria within the tumor not only contributes to the suppression of antitumor immunity and the deactivation of chemotherapeutic drugs [7,16], but also plays a decisive role in the initiation of metastasis [14]. Understanding which microbial taxa are associated with an unfavorable prognosis, how they interact with host cells, and whether they can be controlled opens fundamentally new avenues for the development of diagnostic, prognostic, and therapeutic approaches [17,18,19].

Our results showed low bacterial content in PDAC samples, minimal differences in microbial composition between normal tissue and tumors, no differences in alpha- or beta-diversity, and no correlation between the other clinicopathological characteristics of the tumor and the composition of the intratumoral microbiota. The lack of differences in alpha- and beta-diversity is not surprising. Alpha- and beta-diversity metrics are global and resistant to changes in individual taxa. The absence of significant differences means that the general structure and “geography” of communities and their internal wealth are on average similar between groups. Differential analysis (ANCOM-BC) works at the level of individual taxa. It revealed subtle but statistically significant shifts in the content of specific bacteria, which can be blurred when looking at the entire community.

Given the extremely low microbial biomass of these samples, we were concerned that a significant portion of the detected microbiota was the result of contamination. However, to account for the influence of contamination, we used controls: DNA extraction, blank PCR, sequencing runs, blank paraffin blocks, and swabs from the surfaces used. Ensuring the reliability of conclusions with low bacterial DNA yield from pancreatic samples (FFPE) required strict control at all stages. We combined experimental measures (multiplex PCR, controls) with bioinformatic contaminant filtering (decontam) and advanced statistical methods robust to small datasets (nonparametric tests, ANCOM-BC with FDR correction). This allowed us to exclude the influence of technical artifacts on the final results. The main reasons for the low bacterial yield are likely to be either extremely low initial microbial biomass in the tissue or DNA degradation during long-term storage.

Interestingly, despite the extremely low overall bacterial load in our sample set (consistent with the studies by A. Gihawi et al. [20]), we observed a statistically significant enrichment of *Kocuria rosea* in tumors compared to normal tissue. This result persisted after contaminant filtering using decontam. *Kocuria rosea* is a Gram-positive bacterium that is normally part of the human skin and mucosal microbiota. In a healthy organism, it is considered a harmless commensal [21]. However, *K. rosea* has the ability to become an opportunistic pathogen in immunocompromised individuals [22], and one strain has been reported to produce an exopolysaccharide called cocuran, which has been associated with immunosuppression and inhibition of cell proliferation [23,24]. If *K. rosea* is found more often in tumor tissue, the most likely interpretation is that it is responding to the altered tumor niche rather than driving cancer itself. Tumors can change oxygenation, inflammation, nutrient availability, and immune pressure, which can allow unusual bacteria to persist or expand. A differential abundance of *K. rosea* does not by itself prove causation. It is better interpreted as a marker of ecological change in the tissue, unless the study also shows consistent enrichment, functional effects, and mechanistic evidence linking that organism to tumor biology. However, an artifactual origin of this signal cannot be completely ruled out, given the ubiquitous environmental distribution of *K. rosea* and the well-known difficulties in interpreting sequencing data from ultra-low-biomass samples (stochastic noise, index hopping). Our work emphasizes that the detection of single taxonomic differences under low-biomass conditions must be supported by alternative validation methods before conclusions about the biological role of such microorganisms can be drawn. Thus, while *K. rosea* emerges as a candidate taxon associated with PDAC, its biological significance remains uncertain without orthogonal validation.

Although bacterial composition did not differ significantly between metastatic and non-metastatic PDAC tumors, species-level analysis uncovered distinct patterns relative to adjacent normal tissue. Metastatic tumors exhibited altered abundance of *Kocuria rosea* and Herbaspirillum huttiense, while non-metastatic tumors featured differences in *Lysobacter bugurensis*, *Caulobacter ginsengisoli*, and *Herbaspirillum huttiense*. These findings imply subtle, niche-specific microbial shifts during PDAC progression that evade detection by higher-level taxonomic profiling.

The shared enrichment of *H. huttiense* in both metastatic and non-metastatic tumors suggests a PDAC-associated signature independent of metastatic status, whereas the other species may reflect stage-specific ecological niches. However, given their low abundance and environmental ubiquity, these findings should be considered hypothesis-generating. *H. huttiense* rarely causes human infections; reported cases include severe community-acquired pneumonia and bacteremia in an immunocompetent U.S. adult [25], septicemia in Korea [26], bacteremia in China [27], and bacteremia with infective endocarditis in a pediatric oncology patient [28].

*Lysobacter* was notably enriched in healthy tissues in patients with gastric cancer [29,30], played a central role in liver cancer co-abundance networks [31], declined in breast cancer—where its peptides disrupt cell walls/membranes and promote dysbiosis [32,33]—and correlated positively with NSE expression in lung cancer [34]. Caulobacter has also been linked to gallbladder cancer [35] and PDAC [36]. Given their low abundance and environmental origins, these taxa require validation in independent cohorts and functional assays to distinguish true biological associations from potential contamination.

Similarly to the overall analysis, KRAS-mutant tumors showed differential abundance of *K. rosea* and *L. bugurensis* compared to adjacent normal tissue, but no difference versus wild-type tumors. This suggests that KRAS-driven tumors may not establish a distinct broad microbial signature relative to wild-type tumors, but they do exhibit microenvironmental changes that alter the local bacterial community compared with non-tumor tissue. Such changes may reflect KRAS-associated alterations in tissue metabolism, inflammation, hypoxia, and stromal remodeling, which can create selective conditions for specific low-abundance taxa. Notably, the absence of a difference between wild-type and KRAS-mutant tumors indicates that KRAS status alone may be insufficient to shape the tumor microbiome at higher taxonomic levels, whereas tumor-associated versus normal tissue comparisons are more sensitive to local ecological shifts.

A major strength of this study is the integration of multiple contamination-control strategies—including extensive negative controls (surface swabs, blank paraffin, reagent controls), multiplex PCR optimized for degraded FFPE DNA, and computational decontamination with decontam. This approach, rarely applied at this scale in PDAC microbiome research, provides high confidence that the reported bacterial signals are tumor-associated rather than environmental contaminants. Despite its rigorous approach, the study has a number of limitations. First, archival FFPE tissue introduces DNA fragmentation and variable block age (range not recorded), reducing library yield and potentially biasing detection of low-abundance taxa. Second, sequencing depth varied across samples; while ANCOM-BC is robust to library size, depth-related artifacts cannot be fully excluded. Third, patient treatment history (neoadjuvant chemotherapy, radiation, antibiotic use) was unavailable—these factors alter the tumor microbiome and could confound associations with metastasis or KRAS status. Fourth, tumor stage was not included as a covariate due to insufficient statistical power for multivariate modeling, especially given the small KRAS wild-type subgroup (n = 7, representing ~10% of PDAC). Fifth, the decontamination method may remove environmentally ubiquitous bacteria that are truly present in tissue. Sixth, single-site sampling of each tumor and normal tissue does not capture intratumoral or intrapancreatic microbial heterogeneity. Finally, the small number of M1 cases (n = 3) precludes subgroup analysis of distant metastases. Accordingly, all findings are exploratory and hypothesis-generating, requiring orthogonal validation (qPCR, FISH, culture) and larger prospective cohorts.

## 4. Materials and Methods

### 4.1. DNA Extraction

A collection of FFPE samples of tumor tissues from patients with pancreatic adenocarcinoma and adjacent normal tissues was assembled at the Vishnevsky Institute of Surgery between 2014 and 2018 (Table 1). The study was approved by the ethics committee of the Vishnevsky Institute of Surgery and conducted in accordance with the Declaration of Helsinki. All materials were collected and characterized by the organization’s pathological anatomy department in accordance with the WHO classification of tumors of the pancreas [37]. Each tumor sample contained at least 70% of tumor cells.

To rule out lab contamination, we used several types of negative controls: samples from different surfaces and equipment in the pathological anatomy and molecular genetics labs, 159 paraffin controls (paraffin areas without tissue), and controls from the DNA extraction, PCR, and purification (no template) stages.

DNA was extracted using an ExtractDNA FFPE Isolation Kit (Evrogen, Moscow, Russia) according to the manufacturer’s instructions. DNA quantification was performed using a Qubit 2.0 Fluorometer (Thermo Fisher Scientific, Waltham, MA, USA) with QuDye^®^ dsDNA HS kit (Lumiprobe, Moscow, Russia).

### 4.2. Detection of KRAS Mutations

*KRAS* mutation profiling was conducted on custom biochips as per Emelyanova et al. [38]. Oligonucleotides for immobilization were synthesized (394 DNA/RNA Synthesizer, Applied Biosystems, Foster City, CA, USA) with a 3′-amino modifier (Glen Research, Sterling, VA, USA) using standard phosphoramidite chemistry. Biochips were manufactured by photoinduced copolymerization in a polyacrylamide gel [39]. LNA-containing probes were designed using Exiqon’s software v.10. Target KRAS sequences were amplified via two-step multiplex PCR (SynTaq polymerase, Syntol, Moscow, Russia). Fluorescence was detected using a portable biochip analyzer (Biochip_IMB, Moscow, Russia) with a 100–500 ms exposure [40]. Each probe was spotted in duplicate, and the mean intensity was used for analysis. A signal was considered positive if its intensity exceeded the background by a factor of five, with mutation calls based on the specific pattern of probe hybridization.

### 4.3. 16S rRNA Gene Amplification

The study employed a multiplexed 16S rDNA sequencing protocol that targets five short 16S regions (V2, V3, V5, V6, V8) to increase the coverage and resolution of bacterial species detection from FFPE-derived DNA (fragment length 250–400 bp) [41]. Five regions of the 16S rRNA gene were amplified using 100 ng DNA as an input and a set of 10 multiplexed primers (0.2 μM each primer, F1-TGGCGAACGGGTGAGTAA, F2-ACTCCTACGGGAGGCAGC, F3-GTGTAGCGGTGRAATGCG, F4-GGAGCATGTGGWTTAATTCGA, F5–10 GGAGGAAGGTGGGGATGAC, R1-AGACGTGTGCTCTTCCGATCTCCGTGTCTCAGTCCCARTG, R2-AGACGTGTGCTCTTCCGATCTGTATTACCGCGGCTGCTG, R3-AGACGTGTGCTCTTCCGATCTCCCGTCAATTCMTTTGAGTT, R4-AGACGTGTGCTCTTCCGATCTCGTTGCGGGACTTAACCC, R5-AGACGTGTGCTCTTCCGATCTAAGGCCCGGGAACGTATT), and 2X MULTIPLEX TCR PCR Mix (Milaboratory, Moscow, Russia). Amplification was done with an initial heating step of 95 °C for 5 min, 30 cycles of 30 s at 94 °C, 90 s at 62 °C, and 35 s at 72 °C followed by a final elongation step of 5 min at 72 °C. Barcodes and Illumina adaptors were added to the amplicon with a second PCR reaction with 5 forward primers (0.2 μM each primer, FF1-AATGATACGGCGACCACCGAGATCTACACTCTTTCCCTACACGACGCTCTTCCGATCTTGGCGAACGG20 GTGAGTAA, FF2-AATGATACGGCGACCACCGAGATCTACACTCTTTCCCTACACGACGCTCTTCCGATCTACTCCTACGGGAGGCAGC, FF3-AATGATACGGCGACCACCGAGATCTACACTCTTTCCCTACACGACGCTCTTCCGATCTGTGTAGCGGTGRAATGCG, FF4–25 AATGATACGGCGACCACCGAGATCTACACTCTTTCCCTACACGACGCTCTTCCGATCTGGAGCATGTGGWTTAATTCGA, FF5-AATGATACGGCGACCACCGAGATCTACACTCTTTCCCTACACGACGCTCTTCCGATCTGGAGGAAGGTGGGGATGAC) and one 8-nucleotide barcode-specific reverse primer (0.4 μM, RR5-CAAGCAGAAGACGGCATACGAGAT-NNNNNNNN-GTGACTGGAGTTCAGACGTGTGCTCTTCCGATCT). The amplicon was diluted into the reaction (10-fold) and amplified with 6 cycles of 10 s at 98 °C, 15 s at 64 °C, and 25 s at 72 °C. Libraries were purified from primer dimers using MagPure A4 XP Beads (Magen, Guangzhou, China) at a volume ratio of 1:0.85 (library:beads). Then libraries were combined into the final library (100–430 amplicons) and sequenced on SURFSeq 5000 (GeneMind, Shenzhen, China). Mean sequencing depth was ~27,000 reads for samples and 29,000 for controls.

### 4.4. 16S Analysis

Reads were demultiplexed and processed with the Nextflow 26.04.0 [42] nf-core 4.0.2 [43] ampliseq pipeline v.2.15.0 [44]. Briefly, the following was done. Quality filtering, read pair merging, and amplicon sequence variant (ASV) resolution were performed with DADA2 [45]. Scaffolding of 5R regions and subsequent taxonomic assignment were performed with Silva 138.2 reference [46] using the qiime sidle package [47]. The resulting reconstructed 5R-ASV table was used for the downstream analysis.

Taxonomic features were preprocessed to remove artifactual and non-biological signals. First, ASVs (Amplicon Sequence Variants) were discarded if they contained ambiguous taxonomic annotations. Second, contaminants were identified and removed in a two-step procedure. Any taxon detected in reagent-only or swab extraction controls (prevalence > 0) was excluded. Finally, ASVs were decontaminated with the decontam v.1.30.0 package [17] by using the prevalence method, with a threshold of 0.5 for maximum sensitivity. Differential abundance of remaining taxa was assessed using the ancombc2 function from the ANCOMBC v.2.12.0 package [48]. Alpha- and beta-diversity measures were obtained with the phyloseq v.1.54.0 [49] package. All images were produced using ggplot2 v.4.0.1.

## 5. Conclusions

PDAC tumors harbor very low bacterial biomass, and the microbiome does not robustly distinguish tumor from normal tissue at the community level. However, *K. rosea* emerges as a candidate taxon differentially enriched in PDAC, with potential stage- and KRAS-associated patterns. These findings highlight the need for orthogonal validation (qPCR, FISH, culture) and larger prospective cohorts to differentiate true biological associations from residual contamination or stochastic noise in low-biomass settings.

## Figures and Tables

**Table 1:** Clinical and pathological characteristics of patients with PDAC.

Characteristics	n
Total	50
with lymph node metastases	38
without metastases to the lymph nodes	12
with distant metastases	3
Sex	
female	26
male	24
Age at the time of diagnosis of the disease	
>40	49
<40	1
Grade	
G1	6
G2	26
G3	16
G4	2
Invasion of the resection margin	
R0	26
R1	22
R2	2
Invasion of the lymph nodes	
L0	2
L1	48
Perineural growth	
Pn0	2
Pn1	48
Venous invasion	
V0	16
V1	34
RAS mutation	
WT	7
KRAS61, Q61H	1
KRAS12, G12D	18
KRAS12 G12V	14
KRAS12, G12R	9
KRAS12 G12S	1

**Table 2:** The list of contaminant species found in the negative control samples.

*Brachybacterium conglomeratum*, *Microbacterium maritypicum*, *Veillonella dispar*, *Neisseria subflava*, *Rothia dentocariosa*, *Finegoldia magna*, *Pseudomonas putida*, *Granulicatella elegans*, *Acinetobacter ursingii*, *Corynebacterium suicordis*, *Lactococcus lactis*, *Aerococcus viridans*, *Acinetobacter lwoffii*, *Kingella denitrificans*, *Corynebacterium amycolatum*	Surface swab
*Leuconostoc pseudomesenteroides*, *Haemophilus parainfluenzae*, *Chloroplast rigescens*, *Lactococcus lactis*, *Modestobacter multiseptatus*, *Serratia marcescens*, *Streptococcus salivarius*, *Pelomonas puraquae*, *Streptococcus gordonii*, *Modestobacter Geodermatophilus*, *Micrococcus lylae*, *Streptococcus mitis*, *Staphylococcus aureus*, *Corynebacterium kroppenstedtii*, *Streptococcus infantis*, *Corynebacterium urealyticum*, *Microbacterium chocolatum*, *Corynebacterium simulans*, *Corynebacterium striatum*, *Enhydrobacter integrifolia*, *Rhodococcus corynebacterioides*, *Enhydrobacter osloensis*, *Kocuria palustris*, *Lactococcus delbrueckii*, *Leifsonia xyli*, *Serratia marcescens*	Paraffin
*Pelomonas puraquae*, *Curtobacterium herbarum*, *Herbiconiux flava*, *Aquabacterium commune*, *Bradyrhizobium japonicum*, *Curvibacter lanceolatus*, *Geobacillus toebii*, *Lactobacillus acidophilus*	Reagents
*Propionibacterium acnes*, *Microbacterium chocolatum*, *Corynebacterium tuberculostearicum*, *Cutibacterium melanogenum*, *Chloroplast indica*, *Ralstonia insidiosa*, *Serratia cloacae*, *Acinetobacter junii*, *Microbacterium laevaniformans*, *Tetragenococcus halophilus*, *Gordonia polyisoprenivorans*, *Chloroplast sinensis*, *Staphylococcus epidermidis*, *Caulobacter henricii*, *Brevundimonas diminuta*, *Haemophilus parainfluenzae*, *Variovorax paradoxus*, *Staphylococcus aureus*, *Lawsonella oculatus*, *Staphylococcus haemolyticus*, *Actinomyces naeslundii*, *Streptococcus oralis*, *Curtobacterium flaccumfaciens*, *Peptoniphilus asaccharolyticus*, *Streptococcus cristatus*, *Streptococcus pneumoniae*, *Aquabacterium olei*, *Ralstonia pickettii*, *Serratia marcescens*, *Corynebacterium mycetoides*	Surface swab, reagents

## Data Availability

All data generated or analyzed during this study are included in this published article. The 16S rRNA gene-targeted sequencing are available in the NCBI SRA under the accession number.

## Supplementary Materials

The following supporting information can be downloaded at: https://www.mdpi.com/article/10.3390/ijms27104210/s1.

## Abbreviation

The following abbreviation is used in this manuscript:

PDAC	Pancreatic ductal adenocarcinoma
