# Supplementary figures and images for: Intratumoral Microbiome of Metastatic Pancreatic Ductal Adenocarcinoma
Source: Int J Mol Sci. 2026 May 9;27(10):4210. doi: 10.3390/ijms27104210 (PMC13207053; doi:10.3390/ijms27104210)

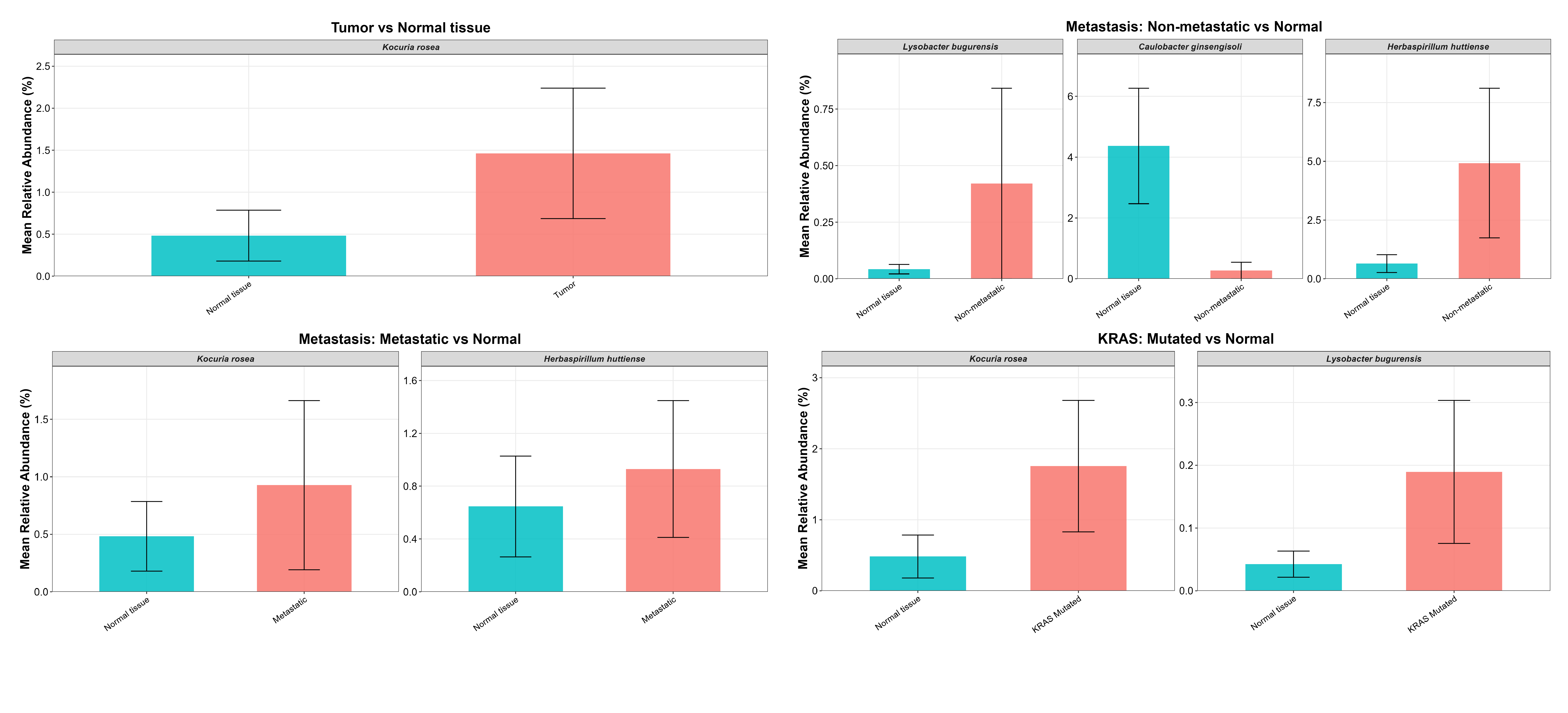

Supplement: Supplementary file 1 [file ijms-27-04210-s001.zip › Supplementary Figure S1.png]
